# Molecularly engineered aspartic acid-intercalated graphene oxide-based membranes for ultrafast removal of dyes and heavy metal salts

**DOI:** 10.1039/d6ra08036g

**Published:** 2026-10-20

**Authors:** Gada Hussain, Sajid Hussain Siyal, Ashique Hussain Jatoi, Khalid Hussain Thebo, Akbar Ali, Ahmed Nadeem, Gamal A. Shazly

**Affiliations:** a School of Material Science & Engineering, Beijing University of Chemical Technology Beijing 100029 China; b Széchenyi István University Egyetem Tér 1 9026 Győr Hungary; c Department of Metallurgy and Materials Engineering, Dawood University of Engineering and Technology Karachi 74800 Pakistan; d Department of Chemistry, Shaheed Benazir Bhutto University Shaheed Benazirabad (67480) Pakistan; e Department of Chemistry, Mirpur University of Science and Technology Mirpur Azad Jammu and Kashmir Pakistan khalidthebo@yahoo.com; f MIIT Key Laboratory of Critical Materials Technology for New Energy Conversion and Storage, State Key Laboratory of Urban Water Resource and Environment, School of Chemistry and Chemical Engineering, Harbin Institute of Technology Harbin 150001 PR China; g Department of Pharmacology and Toxicology, College of Pharmacy, King Saud University Riyadh 11451 Saudi Arabia; h Department of Pharmaceutics, College of Pharmacy, King Saud University Riyadh 11451 Saudi Arabia

## Abstract

Graphene oxide-based composite membranes with controlled nanochannels are effective for the separation of dyes, biomolecules, salts, and heavy metal ions. In this work, aspartic acid@graphene oxide (AA@GO) membranes were prepared by surface functionalization of GO nanosheets with aspartic acid. The AA@GO-0.7% membrane, with a thickness of 320 ± 10 nm, showed excellent rejection of 99.9%, 98.5%, and 99.8% for Rhodamine B, Evan blue, and methylene blue dyes, along with water permeances of 730 ± 10, 815 ± 10, and 800 ± 10 L m^−2^ h^−1^ bar^−1^, respectively. This membrane also demonstrated >98% rejection efficiency for heavy metal salts, *i.e.*, Pb^2+^, Cd^2+^, and As^3+^ ions. It is more interesting that the ultrathin AA@GO membrane (300 nm) demonstrated an exceptionally high water permeance of 1400 ± 10 L m^−2^ h^−1^ bar^−1^. Moreover, aspartic acid intercalation provides an excellent aqueous stability to the AA@GO membrane up to 55 days. These results suggest that AA@GO membrane can be future candidates for advanced water purification system and waste-water treatment applications due to its excellent rejection, high permeability, and enhanced aqueous stability.

## Abstract 2

Herein, aspartic acid-functionalized graphene oxide membranes were prepared. The membranes exhibited excellent separation performance, achieving >99.8% rejection of dye molecules and >98% rejection of heavy metal salts.

## Graphical abstract

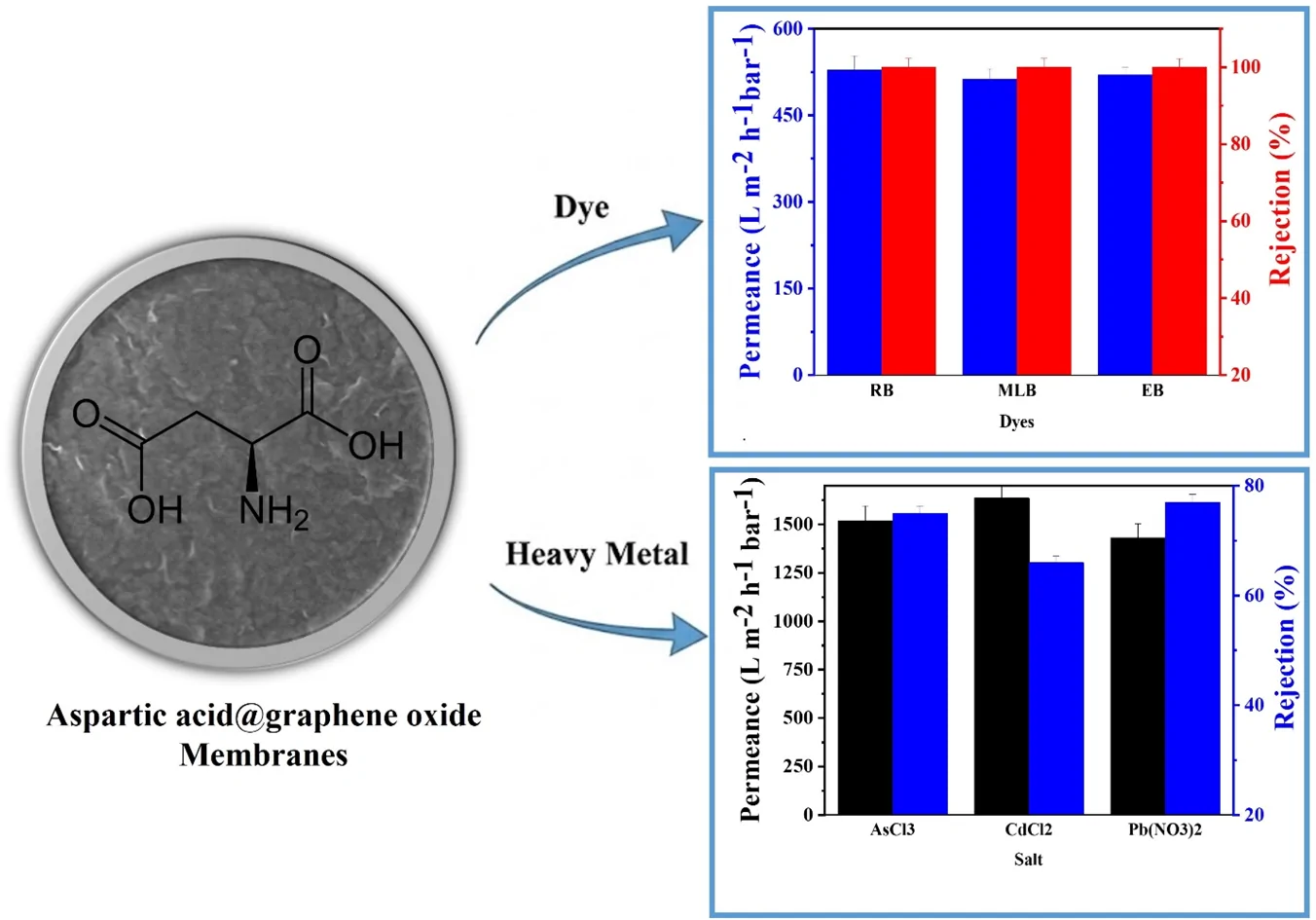

## 1. Introduction

Water pollution caused by the release of synthetic dyes and harmful heavy metals is increasing significantly due to population expansion, increasing industrialization and urbanization, and economic expansion.^1–5^ The entry of these untreated industrial wastes into the ecosystem has contributed to an increased risk of freshwater pollution. Several textile industries release large quantities of synthetic dyes into the environment annually, which produce more than half (54%) of the existing dye wastewater worldwide: the dyeing industry (21%), the paint and leather industry (8%), the pulp and paper industry (10%), and the dye preparation industry (7%).^6–8^ However, the treatment of these dyes is a challenging task for the textile sector due to the complexity of dye molecules and their resistance to heat, aerobic digestion, light, *etc.*,.^9–11^ On the other hand, water pollution caused by heavy metal ions, such as iron, arsenic, copper, zinc, lead, chromium, cadmium, *etc.*, is more critical than dye separation and is the greatest challenge for existing separation technologies.^12–15^ Various industrial processes, including electroplating and metal polishing, discharge these metal ions into water, where they can accumulate in organisms within the food chain. Due to their physicochemical properties, such as high dispersion, small size, and difficult degradation, they have severe impacts on human health and ecosystems.^16–18^ Therefore, economical, sustainable, and effective treatment technologies are required to solve these issues.

Besides conventional water-treatment and separation technologies, membrane-based separation has offered the best solution for overcoming these issues due to its reliability, low cost, simplicity, high efficiency, and environmental friendliness. In this regard, various inorganic and organic materials have been fabricated into membranes for the effective separation of synthetic dyes and heavy metal ions from water. However, these materials cannot achieve success due to their heavy cost, low stability, recyclability, and other fouling issues. Recently, graphene oxide (GO)-based two-dimensional (2D) material has been investigated for selective separation of various dyes, toxic pollutants, heavy metals, and biomolecules because of its low synthesis cost, ease of modification, stability, ultrathin thickness, *etc.*^19–22^ The presence of hydroxyl, epoxide, and carboxyl functional groups on the edges and basal plane of GO makes it have more attractive properties, such as its large lateral dimension-to-thickness ratio, high mechanical strength, and amphiphilic nature.^18,23,24^ Due to these properties, such material remains of keen interest to the scientific community for water treatment and other separation applications. However, water molecules interact with GO nanosheets and increase the interlayer spacing of GO nanosheets up to a few nanometers, and ultimately suppressing the rejection efficiency as well as the stability of membranes during filtration.^25,26^ Therefore, suitable modifications, such as intercalation, crosslinking, functionalization, and composites, shall be required to overcome these issues.^27–30^

Herein, GO nanosheets were intercalated with aspartic acid and fabricated into AA@GO using a vacuum filtration method. Membranes with variable thicknesses were prepared by controlling the concentration ratio of the AA@GO nanomaterials. Aspartic acid intercalation not only increases the interlayer spacing of membranes but also acts as an excellent sieving agent to enhance ion and molecular efficiency. Interestingly, AA@GO-0.7% (320 ± 10 nm) membranes showed 99.9%, 98.5%, and 99.8% rejection efficiency of RB, EB, and MLB dyes, along with 730 ± 10, 815 ± 10, and 800 ± 10 water permeances, respectively. Furthermore, such a membrane also exhibited good rejection efficiency (∼98%) of As^3+^, Cd^2+^, and Pb^2+^ ions. Additionally, a 1330 nm thick AA@GO membrane also exhibited excellent aqueous stability for up to 55 days. These results demonstrate the potential of AA@GO membranes as efficient and stable materials for water purification, waste-water treatment, desalination, and other selective separation applications.

## 2. Experimental

### 2.1. Materials

The chemicals, such as graphite flakes, sulfuric acid (95–98% H_2_SO_4_), sodium nitrate (∼99%), and potassium permanganate (∼99%), used in the synthesis of GO nanosheets, were procured from Sigma-Aldrich, China. The aspartic acid was purchased from Sigma-Aldrich, China and utilized for intercalation of the GO nanosheets. While nylon filter support (pore size 0.45 µm, diameter 50 cm) was used as support membrane for fabrication of GO-based membranes.

### 2.2. Preparation of GO nanosheets

The GO nanosheets are synthesized according to the reported method.^31^ First, 6.0 g of 325-mesh graphite flakes and 4.5 g of NaNO_3_ were stirred with 288 mL of concentrated sulfuric acid in an ice bath with constant magnetic stirring at 20 °C. Then, 27 g of KMnO_4_ was mixed with the reaction mixture and further stirred at below 10 °C. After that, the mixture was stirred for 90 min below 20 °C in an ice bath, and then for an additional two hours at 35 °C with constant magnetic stirring. Further, 138 mL of DI water was added dropwise to the reaction mixture to avoid heating. After that, 420 mL of DI water and 9 mL of 30% H_2_O_2_ were added with continuous stirring to synthesize graphite oxide suspension. The as-synthesized product was washed several times with acidic and DI water until a pH of near 6 was obtained using a centrifuge machine (20 000 rpm, 10 min). After that, the suspension was dialyzed for up to three days to remove metallic contamination. Finally, graphite oxide was exfoliated into GO dispersion with the help of tip sonication (1 h and 135 W). Then, the GO dispersion was collected *via* centrifugation (9000 rpm for 10 min) to remove large multilayer flakes. The as-prepared product was dried and used for further membrane preparation.

### 2.3. Preparation of the AA@GO composite materials

The GO nanosheets (0.15 g) were dispersed into 100 mL of DI water using tip sonication. Then, 0.15 g of aspartic acid was added to the GO dispersion. The obtained reaction mixture was heated at 80 °C in a closed round-bottom flask with continuous stirring for 24 h. Finally, a dark black composite of AA@GO was prepared (AA@GO-0.15). The as-prepared AA@GO-0.15 composite was washed with DI water to avoid contamination. The final product was collected *via* centrifugation and dried at 60 °C for further use. Furthermore, different AA@GO composites were prepared *via* a similar method by varying the amount of aspartic acid (0.30 g, 0.5 g, and 0.7 g) used instead of 0.15 g.

### 2.4. Preparation of AA@GO membranes

Initially, a dispersion solution of the AA@GO-0.15 g composite was prepared by adding 0.15 g of composite to 100 mL of DI water, after which the as-prepared composite was filtered through a nylon (0.45 µm) support using a dead-end filtration device. The as-prepared membrane was dried at room temperature and used for further testing. The thicknesses of the membranes were controlled by adjusting the volume of the dispersion solution (0.15 mL, 3 mL, 6 mL and 8 mL) during the filtration process. Furthermore, composite membranes (AA@GO-0.3, AA@GO-0.5 and AA@GO-0.7) were also prepared by using the same ratios of the various materials.

### 2.5 Characterization of the membranes

Physicochemical characterization of the as-prepared GO and AA@GO membranes was performed using various techniques. We used a D-MAX/2400 XRD diffractometer with Cu K_α_ radiation at a wavelength of 0.154 nm to obtain X-ray diffraction (XRD) patterns. X-ray photoelectron spectroscopy (XPS) was performed using ESCALAB250 equipment with a 150-watt power output and 500-micrometer spot size to identify the elements. The 284.6 eV binding energy of adventitious carbon was used to calibrate all of the spectra. Bruker DektakXT Stylus Profiler (Germany) was used to measure the thicknesses of membranes. A Carry 50 UV/vis spectrometer (Varian USA) was used to measure the absorption of feed and permeate solutions. The thicknesses of all membranes were measured using a Bruker DektakXT Stylus Profiler (Germany) after drying the membranes at room temperature for 24 h. The hydrophilic properties of the membrane surfaces were determined using a contact angle goniometer (USA, F2 Series, ramé-hart) according to standard protocols. The surface and cross-sectional structural morphologies of all membranes were observed with the help a scanning electron microscope (SEM, JOEL JSM-7600F).

### 2.6. Separation performance of membranes

The separation performance, *i.e.*, rejection and permeance, of the as-prepared membranes was measured *via* vacuum filtration assembly and dead-end filtration using different 250 mL of feed solutions at 0.9 bar pressure. Initially, the water permeance was observed every ten minutes until a consistent reading was achieved. Then, the efficiency of membranes was evaluated using 250 mL of feed solution. Prior to the experiment, each membrane was stabilized for 1 h before collection of permeate solution and then measured efficiency and permeance using eqn (1) and (2).1
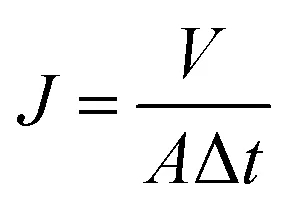
where, *J* is the water permeance, *V* represents the permeate volume (liters) and effective area is represented *A* in square meters (m^2^).2
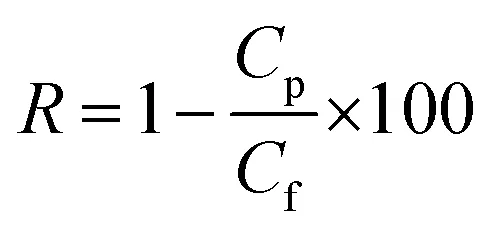
where Δ*t* represents the time in hours, and *C*_p_ and *C*_f_ are the concentrations of the feed and permeate solutions, respectively.

## 3. Stability and swelling studies

As for the stability of membranes, both membranes were cut into small, different-sized pieces and dipped into different pH solutions (pH = 7, 2.4, 12.6). The readings of stability were measured on a daily basis in static mode, and digital photos were taken and used for data.

To further measure the swelling of membranes according to our reported method.^32^ Initially, the weights of dry pGO and AA@GO-based membranes were noted using an analytical weight balance at room temperature. After that, both membranes were dipped in DI water for up to 24 h. Then again, membranes were dried at room temperature for 24 h. At this stage, the weights of membranes were noted before and after drying. Finally, the obtained values were used to calculate the degree of swelling according to the previously reported method.^32^

## 4. Results and discussion

### 4.1. Physicochemical characterization

In this work, GO nanosheets with monolayers thickness 1 nm and lateral size 0.5 to 10.5 µm were synthesized, as shown in Fig. S2. Further, the aspartic acid was used to modify GO nanosheets, which were subsequently fabricated into AA@GO-based membranes (Fig. 1a). The aspartic acid molecule contains a unique combination of amino and hydroxyl groups (Fig. S1) that form hydrogen bonds and electrostatic interactions with the GO surface (Fig. 1a). Furthermore, an SEM study of the pristine GO (pGO) and AA@GO-based composite membranes was carried out to observe the structural morphology of the membrane. The surface and cross-sectional morphology of the pGO and AA@GO composite membranes are shown in Fig. 1a–d, respectively. Fig. 1b and c shows the surface morphology and cross-sectional structure of the pGO membranes, which clearly show the rough surface of the pGO membranes along with their tightly layered structures. On the other hand, the AA@GO composite membrane is smooth and uniform in morphology, and there is a large interlayer spacing between the 2D sheets, as shown in Fig. 1d and e, respectively. The large interlayer spacing between GO nanosheets is possibly due to the successful cross-linking of aspartic acid.

**Fig. 1:**
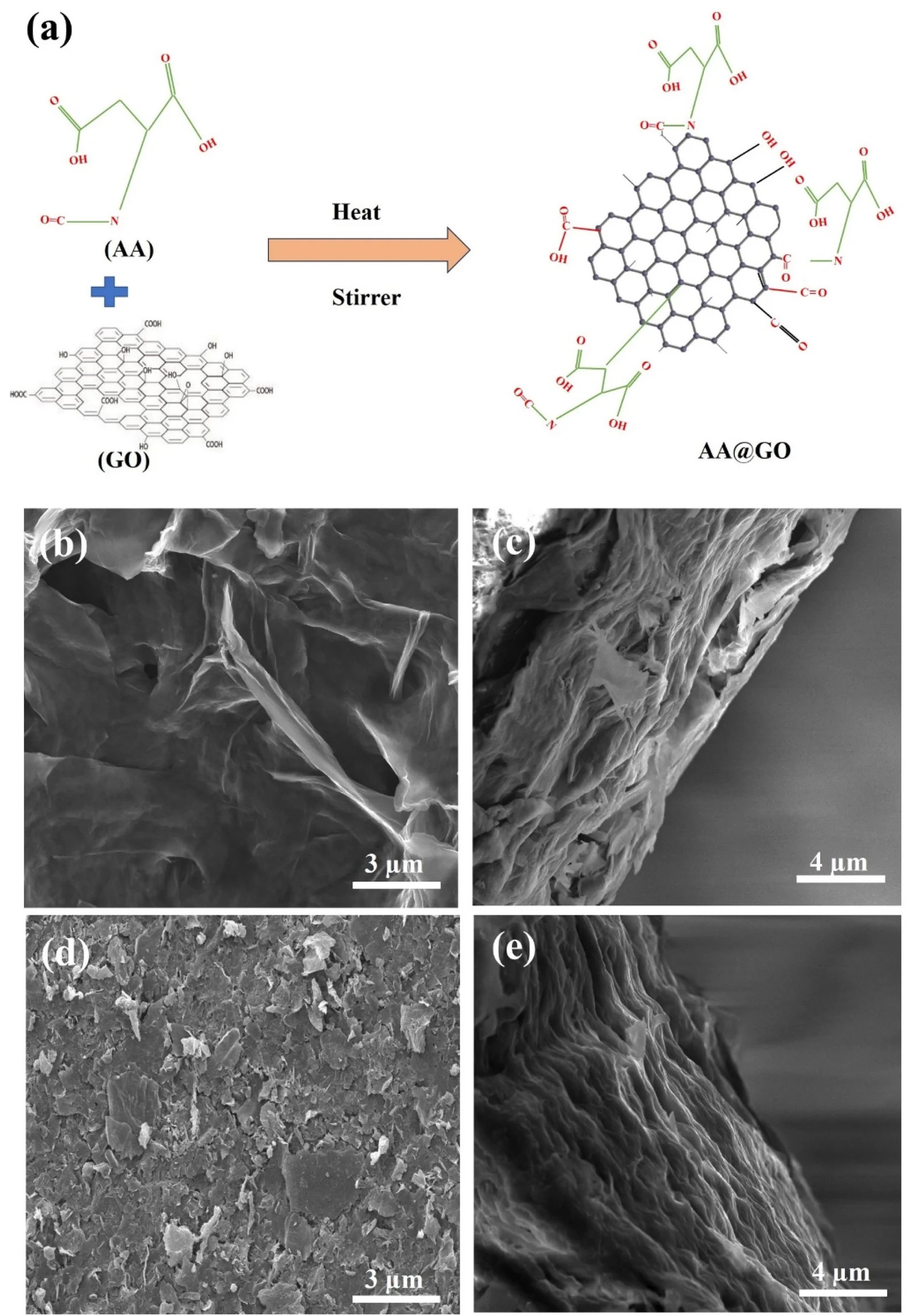
(a) The schematic representation of GO nanosheets with aspartic acid interactions in AA@GO membranes. (b–e) SEM surface and cross-sectional studies of the (b and c) pGO and (d and e) AA@GO membranes, respectively.

Furthermore, the XRD patterns of the GO and AA@GO membranes are shown in Fig. 2a. The XRD peak of GO was observed at 10.166° with an interlayer spacing of 0.87 nm. However, after GO sheets were modified with aspartic acid, the interlayer spacing increased, and an 9.1° peak corresponding to an interlayer spacing of 0.97 nm was obtained for the AA@GO composite. The interlayer distance between 2D nanosheets increases due to the structure, functionality, and chain length of the aspartic acid present within GO sheets.

**Fig. 2:**
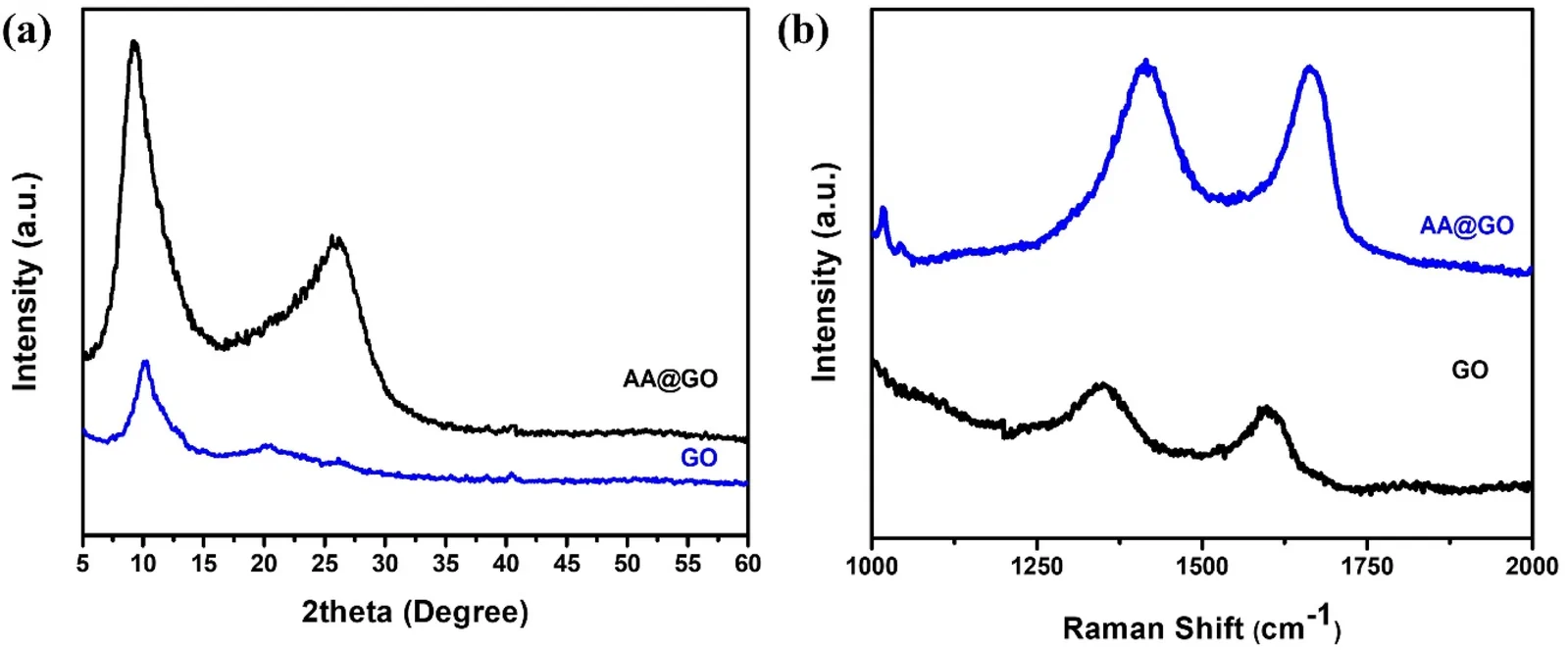
XRD patterns (a) and Raman spectra (b) of the as-synthesized AA@GO and pGO membranes, respectively.

In addition, Raman spectroscopy was utilized to investigate the structural changes after modifying GO nanosheets with aspartic acid (Fig. 2b). The as-synthesized pGO membranes showed two peaks related to the sp^3^ (D-band) and sp^2^ (G-band) carbon atoms. The ratio of the intensity of the D bond to the intensity of the G bond reveals the bond intensity of sp^3^ carbon atoms to sp^2^. In the GO membranes, D and G bonds are observed at 1345 cm^−1^ and 1583 cm^−1^, respectively. However, after cross-linking GO membranes with aspartic acid, the intensity of D and G bonds increased to 1352 cm^−1^ and 1593 cm^−1^, respectively. Therefore, new sp^3^ bonds within GO nanosheets and defects in the structures increased the ID/IG ratio of AA@GO membranes up to 1.02, compared to pGO membranes (0.91).

Furthermore, XPS was performed to validate the properties of the functional groups present in the GO and AA@GO membranes, as shown in Fig. 3. The XPS spectrum of the AA@GO membranes revealed peaks related to C 1s, O 1s and N 1s at 284.9, 531, and 400 eV, respectively (Fig. 3a). Fig. 3b shows the four fitted C 1s deconvoluted curves of the AA@GO membranes, which provides an illustration of the binding energies of C–C/C=C, C–N, and C=O, that are 284.6 eV, 285.8 eV, and 286.9 eV, respectively. However, the peak at 284.2 eV that is observed in the XPS spectra of the GO membranes is a result of the aromatic ring that is present in the GO membranes and corresponds to C=C/C–C. While the signal at 286.2 eV demonstrated the presence of alkoxyl functional groups and 1,2 peroxides, it also confirmed the presence of the former. Additionally, the carbonyl (C=O) signal was observed at 287.3 eV, and the epoxy (–C–O–C–) peak was confirmed to exist at 288.7 eV. Fig. 3b clearly showed that the intensity of the C–O peak drastically decreased after intercalation with aspartic acid compared to pristine GO membranes,^31^ confirming the GO sheets are reduced.

**Fig. 3:**
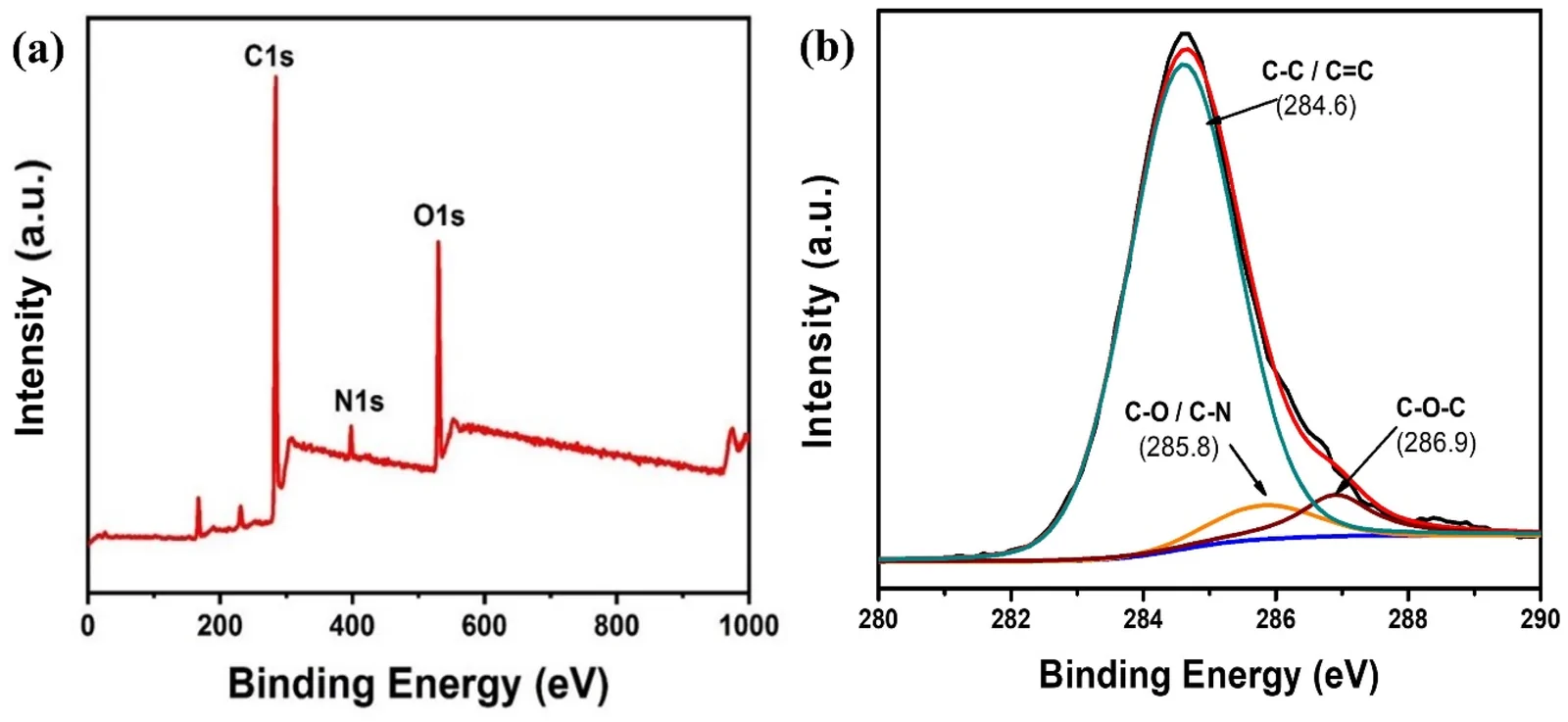
(a and b) XPS studies of the as-synthesized AA@GO membranes. (a) XPS survey and (b) the deconvoluted curve from the C 1s XPS spectra.

Furthermore, the functional groups of the GO and AA@GO composite membranes were also confirmed by FTIR studies (Fig. S3a and b). In the spectrum of the pGO membranes, the broad stretching band corresponding to the hydroxyl (O–H) group was observed at 3240 cm^−1^. However, C–O stretching and C=C stretching of carboxylic groups were observed at 1706 and 1623 cm^−1^, respectively (Fig. S3a). C–O shows a stretching peak at 1042 cm^−1^. On the other hand, the FTIR spectrum of the AA@GO membrane confirmed N–H/O–H stretching at 3267 cm^−1^. The band for C–H stretching was obtained at 2898 cm^−1^, and the C=O stretching/C=C vibration band was observed at 1692 cm^−1^ (Fig. S3b). In addition to this, a vibration band for C–N/O–H was observed at 1344 cm^−1^. The bands at 1163 and 1004 cm^−1^ further indicate the presence of C–O-containing functionalities.

### 4.2. Stability test of membranes

Several studies have demonstrated the lower stability of GO-based membranes in an aqueous environment because of the presence of functionalities.^33–35^ Due to these functional groups, GO membranes delaminate and immediately swell in water. In this regard, various strategies have been investigated to improve the stability and swelling of membranes. Therefore, we investigated the swelling of the pGO and AA@GO membranes (Fig. 4a). The pGO membrane exhibited an equilibrium weight swelling ratio (ESR) of 2.2 ± 0.1. However, this swelling was still reduced to 1.7 ± 01 for the AA@GO0.15% membrane. To better understand the swelling behavior of these membranes, we prepared AA@GO membranes with aspartic acid loading ratios of 0.15%, 0.3%, 0.5%, and 0.7%. Interestingly, the swelling ratios drastically decreased as the aspartic acid ratio increased, and the AA@GO-0.7 composite membrane exhibited low swelling (∼0.7 ± 0.1). This demonstrated that the strong interaction between the aspartic acid molecule and the GO sheet causes the higher aspartic acid ratio to decrease the number of functional groups on the GO sheets.

**Fig. 4:**
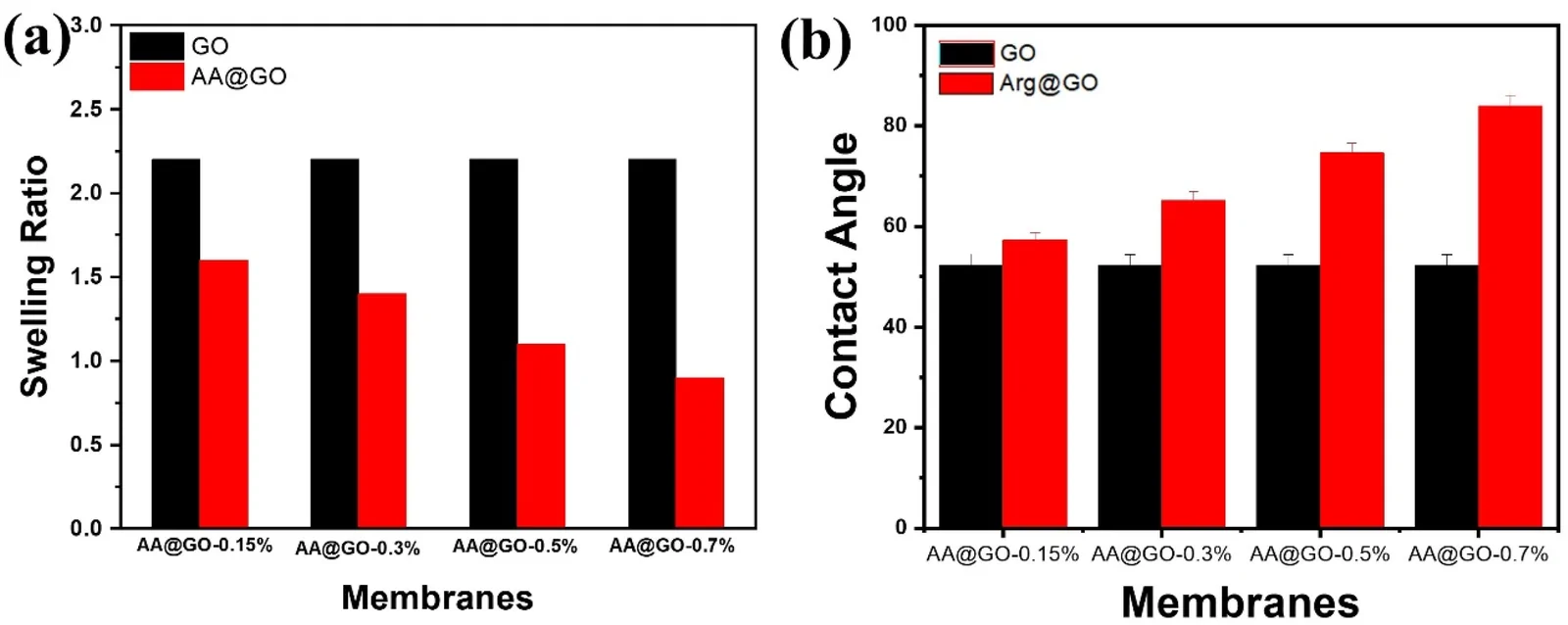
(a) Swelling study ratio and (b) contact angle measurements of 320 ± 10 nm thick GO (black color) and AA@GO composite membranes with different ratios of AA (red color).

Furthermore, we investigated the hydrophilicity and hydrophobicity of AA@GO membranes with different ratios (Fig. 4b). The pGO membrane had a contact angle of 53.2° (Fig. 4b) and exhibited more hydrophilic properties. These properties are due to the presence of additional functional groups on the edges and basal planes of these materials. However, after GO membranes were modified with aspartic acid, the membranes were less hydrophilic and had a contact angle of 59.3° (Fig. 4b), which is possibly due to the reduction of GO sheets. This reduction of GO nanosheets is happened due to the presence of amino groups and effect of temperature (70–80 °C) during the modification process. The hydrophobicity of the AA@GO membranes increased to 84.8 ± 1 when the loading ratio of aspartic acid increased from 0.15% to 0.7%.

In addition, the stability of the pGO and AA@GO membranes was assessed through immersion in solutions with varying pH values (7.0, 2.4, and 12.6). Fig. 5a–c clearly shows that the pGO membranes are delaminated within 2 to 5 days in acidic, basic and neutral solutions. However, our fabricated AA@GO membranes are stable in acidic, basic and neutral media for up to 25 to 55 days (Fig. 5). Such membranes showed high stability in water for up to 55 days (Fig. 5d and e). This high stability in water is attributed to two reasons. First due to presence of amino functional groups of aspartic acid. The amino groups strongly attract the hydroxyl functional groups of GO sheets. As a result, the swelling ratios of membranes are decreased, and the membranes cannot easily delaminate into water. Second, the GO nanosheets are reduced in AA@GO membranes to some extent due to the presence of amino groups within aspartic acid and heat treatment. The stability of the membranes increased several times in water. However, these membranes exhibited decreased stability for up to 25 days in acidic and basic solutions (Fig. 5f–i). The low stability of these membranes is due to the high concentration of hydrogen ions in acidic solutions and the high concentration of hydroxyl ions in basic solutions. However, in an acidic environment, membranes are easily destroyed due to the high concentration of hydrogen ions (H^+^), which weakens the interlayer interaction and is responsible for the delamination of membranes from the substrate. On the other side, in alkaline condition, a high concentration of OH^−^ ions can also increase the hydration of oxygen-containing groups and enhance electrostatic repulsion between adjacent GO sheets, resulting in membrane swelling and, ultimately, delamination.

**Fig. 5:**
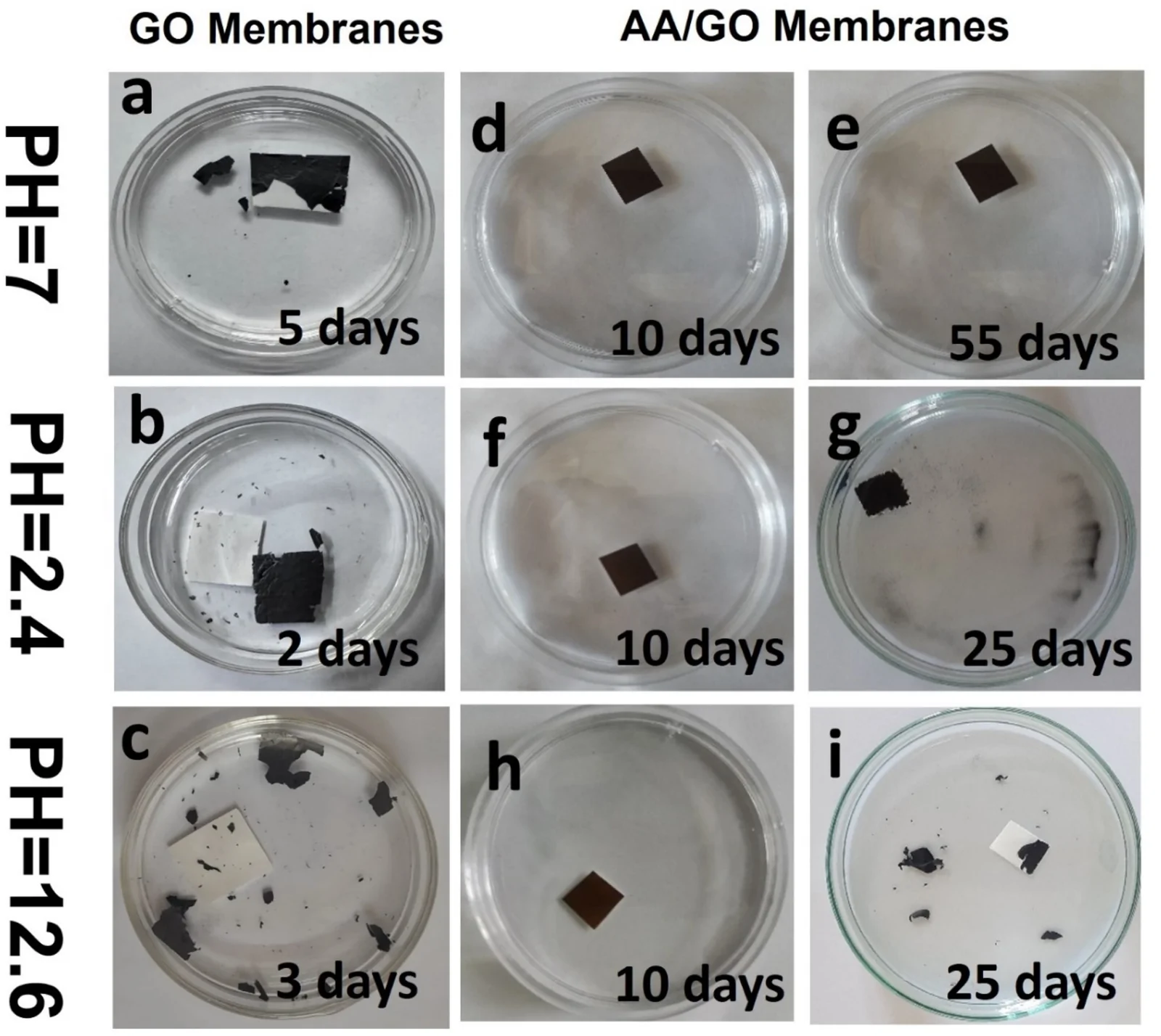
(a–c) Stability of the 320 ± 10 nm thick pGO membrane in (a) DI water at pH = 7, (b) acidic solution at pH 2.4, and (c) basic solution at pH 12.6. (d–i) Stability of 300 ± 10 nm thick AA@GO-0.15% composite membranes in (d and e) DI water at pH = 7, (f and g) acidic solution at pH 2.4, and (h and i) basic solution at pH 12.6 on different days.

### 4.3. Separation performance of the membranes

#### 4.3.1 Water permeance

The pGO membranes were prepared with different thicknesses, such as 320 ± 10, 650 ± 10, 945 ± 10, and 1280 ± 10 nm by controlling the ratio of GO dispersion and were used to measure the water permeance under an applied pressure of 0.9 bar (Fig. 6a). The 320 nm-thick pGO membrane demonstrated a water permeance of 27 ± 1 L m^−2^ h^−1^ bar^−1^ (Fig. 6b). As the thickness of the membrane increased, the water permeance of the membranes decreased drastically, reaching approximately 7.0 L m^−2^ h^−1^ bar^−1^ for the 1280 ± 10 nm membrane. The decrease in permeance is attributed to the close stacking of GO nanosheets and the greater degree of sheet overlap, which increases the effective transport resistance. Furthermore, the water permeances of different AA@GO membranes, *i.e.*, 300 ± 10, 680 ± 10, 970 ± 10, and 1330 ± 10 nm, were measured (Fig. 6b). On the other wise, the as-prepared 300 ± 10 nm-thick AA@GO membrane exhibited a water permeance of 1400 ± 10 L m^−2^ h^−1^ bar^−1^, which is several magnitudes greater than that of the pGO membranes. This superior water permeance of as-prepared AA@GO membranes can be attributed to the large interlayer spacing of GO nanosheets produced by intercalation of aspartic acid between GO nanosheets, which further facilities fast water transport. In addition, we study the effect of thickness of AA@GO membranes on water permeance. Nevertheless, permeance decreased significantly with increasing membrane thickness, and 1330 ± 10 nm-thick AA@GO membrane exhibited water permeance of 315 ± 10 L m^−2^ h^−1^ bar^−1^, due to extended transport pathways and higher flow resistance across the additional GO layers. Ultimately, these findings highlight that both interlayer spacing and thickness critically govern membrane performance, with aspartic acid intercalation providing a substantial enhancement over pristine GO.

**Fig. 6:**
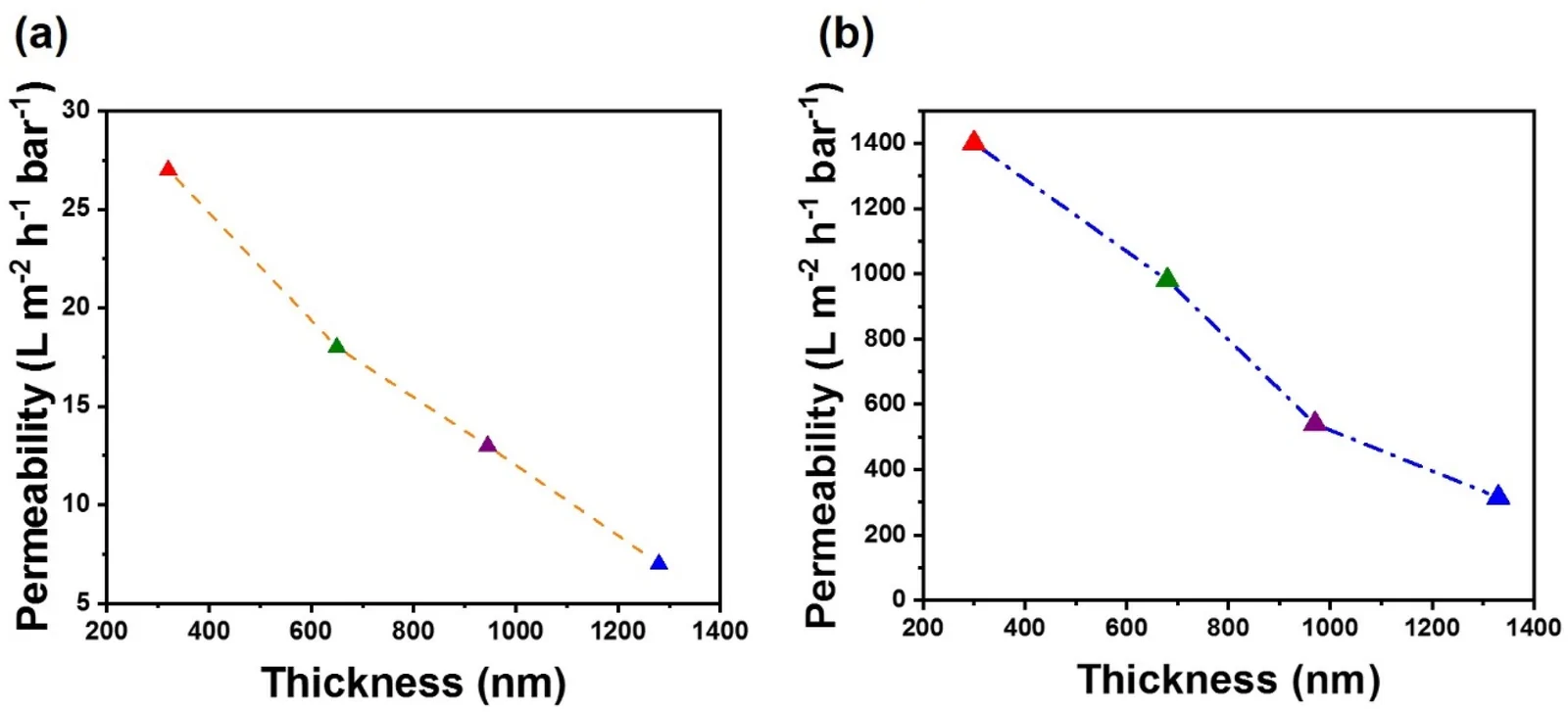
(a) Water permeance performance *versus* thickness of the pGO membranes. (b) Water permeance performance *versus* thickness of AA@GO-0.15% membranes with different thicknesses at a pressure of 1 bar. (AA@GO membranes with a 0.15% loading ratio of AA to GO each).

#### 4.3.2 Rejection efficiency of dye and heavy metal salt

Initially, the rejection efficiency of the 320 ± 10 nm-thick pGO membranes was evaluated using MLB, EB, and RB dyes (Table S2). The confirmation of concentrations of feed solution before and after filtrate was determined with a UV-vis spectrophotometer. The pGO membranes exhibited high rejection efficiencies, reaching 98.2% for EB, although they showed relatively low water permeance. While such membranes showed very low efficiency, *i.e.*, 96.1% and 95.1% for the RB and MLB dyes, respectively. The high rejection of Eb dye is possible due to its large molecular size and more extended molecular structure compared to MLB and RB molecules, which significantly increases its steric hindrance during water transport through the narrow interlayer spacing of the membrane.

Further, rejection efficiency and molecular cutoff of the as-prepared AA@GO membranes were evaluated using RB, MB, and EB dyes, which possess different molecular masses and sizes (Table S1). Previous studies have shown that GO nanosheets can strongly adsorb dye molecules and heavy metal ions owing to their abundant oxygen-containing functional groups.^36^ Therefore, to minimize the contribution of adsorption and obtain a more accurate assessment of the membrane separation performance, the membrane samples were stabilized for 60 min under a low applied pressure of 0.6 bar before collecting the feed and permeate solutions.^22^

The AA@GO-0.2% membrane showed excellent rejection up to 99.9%, 99.8%, and 99.5% for the RB, MLB and EB dyes, respectively, as shown in Fig. 7a. This rejection efficiency is several times greater than that reported GO-membranes (Table S4). Although these membranes have larger interlayer spacing but still, they exhibit a high rejection efficiency for dye molecules. The high rejection is duet to presence of multiple functional groups (carboxyl, hydroxyl and amino). These groups modify the surface charge and chemical environment of the GO nanochannels and promote interactions with dye molecules. Therefore, dye transport is restricted through a combination of molecular sieving, confined/tortuous nanochannels, and electrostatic and hydrogen-bonding interactions, rather than by size exclusion alone. This allows the AA@GO membrane to achieve excellent dye rejection despite its relatively large interlayer spacing.

**Fig. 7:**
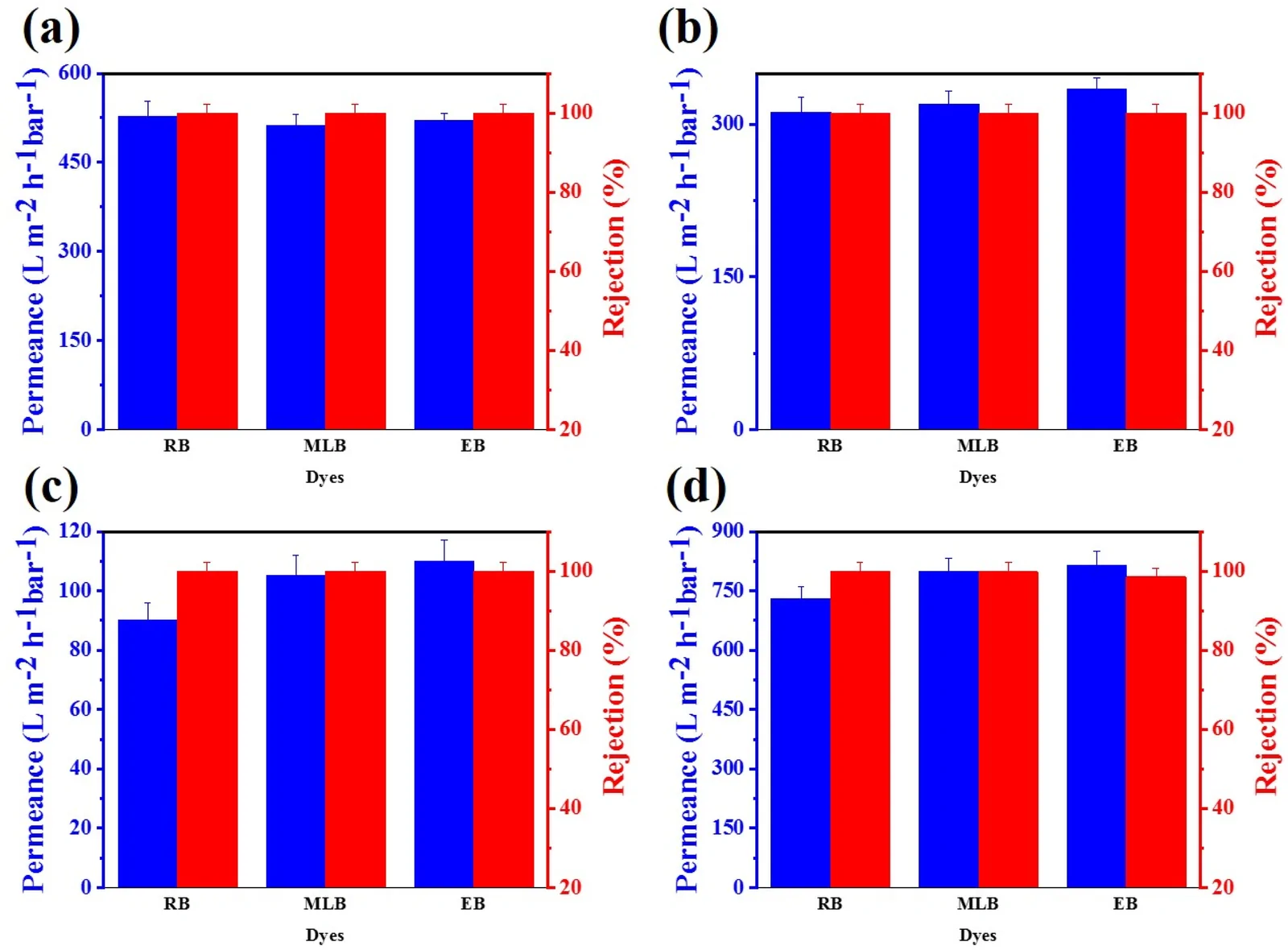
(a–d) Separation performance of the AA@GO membranes against RB, MLB, and EB dyes using different loading ratios of aspartic acid (a) AA@GO-0.15% (300 ± 10 nm), (b) AA@GO-0.3% (310 ± 10 nm), (c) AA@GO-0.5% (305 ± 10 nm), and AA@GO-0.7% (320 ± 10 nm) membranes (the concentration of each dye was 100 ppm).

Further, AA@GO membranes were fabricated with different loading ratios of aspartic acid and evaluated for dye separation, as shown in Fig. 7a–d. As the loading ratio of aspartic acid increased from 0.15% to 0.7%, the rejection efficiency of the MLB, RB, and EB dyes significantly increased (Fig. S4), but the permeance of the membrane decreased gradually (Fig. 7a–d). The increased dye rejection is due to the increasing number of functional groups (amino and carboxyl groups) introduced by aspartic acid, which strengthen with dye molecules and modify the physicochemical environment of the GO nanochannels. In addition, increased aspartic acid incorporation can alter the interlayer structure and create more selective and tortuous transport pathways, thereby enhancing molecular sieving. While decreased water permeance at high aspartic loading may arise from increased transport resistance within the membranes due to greater functionalization, increased pathway tortuosity, and changes in the surface chemistry and interlayer environment. Therefore, we can conclude that aspartic acid plays a dual role in the AA@GO membrane. First, it is providing large interlayer spacing and is responsible for the high water permeance. Second, it works as a molecular sieving agent and accounts for the remarkable rejection efficiency of dyes.

The study showed that the rejection efficiency of dyes is also affected by their sizes, charges, shapes, and molecular weights. In this study, charge and size are important factors governing the separation performance of the AA@GO membranes. The zeta-potential measurements exhibited that the AA@GO membrane surface is positively charged under the investigated conditions. Therefore, highly positively charged surfaces repel cationic dyes with high efficiency, such as RB and MLB dyes (Fig. 7). These finding indicated that AA@GO membranes showed high rejection for large-size molecules compared to small-size molecules, which suggesting steric hindrance and molecular sieving effect of the membranes. Further, the different functional groups (amino, carboxyl, *etc.*) present on the surface of AA@GO membranes can also enhance the rejection efficiency due to their intercalation with feed molecules through hydrogen bonding, electrostatic interaction, and other intermolecular forces. Therefore, the high dye rejection observed for AA@GO membranes is most appropriately attributed to the synergistic effects of molecular sieving, electrostatic interactions, and surface adsorption rather than to a single separation mechanism.

In addition, the filtration performance of AA@GO membranes was investigated using AsCl_3_, CdCl_2_, and Pb(NO_3_)_2_ heavy metal salts, at a concentration of 50 ppm in the feed solution, as shown in Fig. 8 and Table S3. The separation performance of AA@GO membranes with different thicknesses is shown in Fig. 8a–d. First, a 300 nm-thick AA@GO membrane was used to measure the rejection efficiency of metal salts. The membranes showed 75% and 77% rejection for As^3+^ and Pb^2+^ salts, respectively, as shown in Fig. 8a. In comparison, increasing the membrane thickness to 1330 nm of membrane enhanced efficiency up to 98.9%, 99.9%, and 99.9% for AsCl_3_, CdCl_2_, and Pb(NO_3_)_2_, respectively, as shown in Fig. 8d. The observed rejection followed the order CdCl_2_ > AsCl_3_ ∼ Pb(NO_3_)_2_. This high rejection efficiency with increasing thickness of membranes can be due to the increased transport resistance and longer diffusion pathways provided by the AS@GO layers. In addition, the separation of charged metal species is influenced by electrostatic interactions between the ions and the charged functionalized GO nanochannels. The positively charged surface of AA@GO membranes can hinder the transport of positive ions (cations) through electrostatic repulsion. Further, the other functional groups, such as amino, carboxyl, and oxygen-containing functional groups, can interact with heavy metal ions and affect their transport. On the other side, the rejection of divalent metal salts is significantly higher compared to lower-valence ions because of their higher charge density and stronger interaction with these functional groups.^27^ Therefore, it can be concluded that this rejection of metal ions is influenced by a molecular/ionic sieving, ion-functional-group interactions, electrostatic exclusion, and the increased transport resistance associated with the thickness of the membrane. They collectively enable membranes to achieve excellent efficiency of heavy-metal salts, particularly at greater membrane thicknesses.

**Fig. 8:**
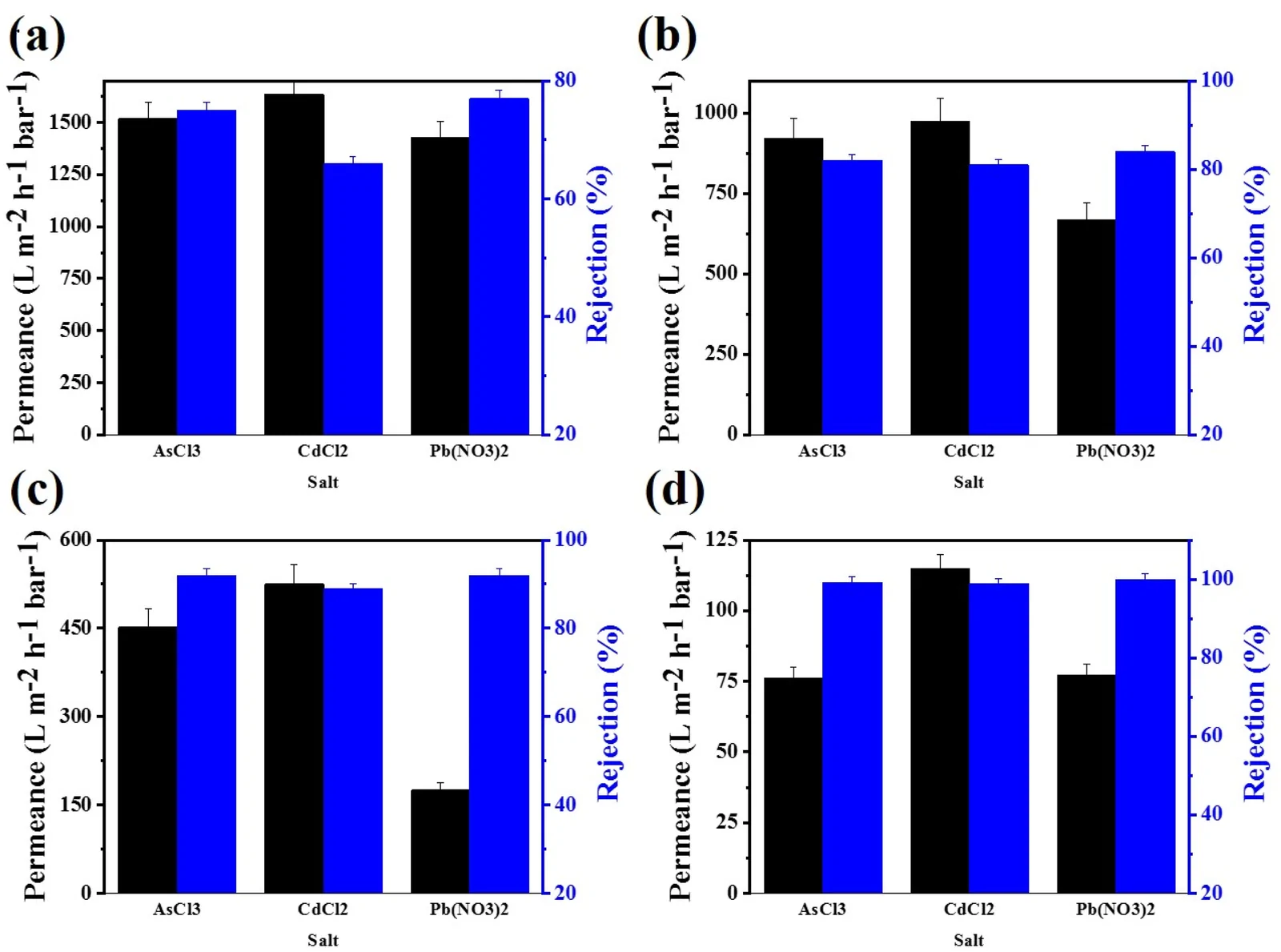
(a–d) Heavy metal salt retention and water permeance of the (a) 300 ± 10 nm, (b) 680 ± 10 nm, (c) 970 ± 10 nm, and (d) 1330 ± 10 nm thick AA@GO-0.15% composite membranes with different thicknesses. (The concentration of each heavy metal salt was 50 ppm).

Moreover, the long-term operational stability and separation efficiency of membranes have been studied, and we have selected RB as a model dye. The separation performance of the AA@GO membrane against RB dye was tested for up to 24 h. However, after 12 h, the AA@GO membrane's rejection efficiency dramatically dropped, and the membrane demonstrated 77 ± 2% rejection. When studies were carried out for 24 h, we observed a significant decrease in rejection efficiency, and the membrane demonstrated 48 ± 2% rejection for RB dye. The low rejection efficiency of the membrane is possibly due to the oversaturation of dyes and interaction with the surface of the membrane. Further, we have evaluated the reusability of our AA@GO membrane using RB dye. Initially, the membrane rejected RB dye (100 ppm) with 99 ± 1% efficiency. However, after the washing process, the membrane showed less rejection, up to 78 ± 1% for RB dye, and delaminated, which is due to its ultrathin thickness. Therefore, it can be concluded that a modification of GO nanosheets with aspartic acid not only improves the stability of the membrane but also has a significant influence on the separation efficiency.

## 5. Conclusion

In this work, GO nanosheets were intercalated with aspartic acid and fabricated into large interlayer spacing membranes using vacuum filtration assembly. Membranes with variable thicknesses were prepared by controlling the concentration ratio of the AA@GO composite materials. This study also showed that aspartic acid intercalation not only increases the interlayer spacing of membranes but also acts as a molecular and ionic sieving agent to enhance efficiency. Interestingly, the 300 nm-thick AA@GO membrane demonstrated an ultrahigh water permeance of 1400 ± 10 L m^−2^ h^−1^ bar^−1^ and had a high rejection efficiency (>99%) of RB, EB, and MLB dyes. Furthermore, such a membrane also exhibited good rejection efficiency (98%) of As^3+^, Cd^2+^, and Pb^2+^ ions. Additionally, these membranes are able to maintain their stability for more than 55 days even when subjected to extreme circumstances, including acidic and basic environments. Further, we have measured the operational stability and separation performance of AA@GO membrane against RB dye, which was tested for up to 24 h, and the membranes showed 48 ± 2% rejection of RB dye even after 24 h. Therefore, these membranes can be an alternative solution for various wastewater treatments and selective separations in the future.

## Conflicts of interest

The authors declare no conflicts of interest related to this research work.

## Supplementary Material

RA-016-D6RA08036G-s001

## Data Availability

Data supporting the finding of this work are available in this article and its supplementary information (SI). Additional datasets used and/or analyzed in this study are available from the corresponding authors upon request. Supplementary information is available. See DOI: https://doi.org/10.1039/d6ra08036g.
