# Supplementary material for: Molecularly engineered aspartic acid-intercalated graphene oxide-based membranes for ultrafast removal of dyes and heavy metal salts
Source: RSC Adv. 2026 Oct 20. Online ahead of print. doi: 10.1039/d6ra08036g (PMC13681188; doi:10.1039/d6ra08036g)
Supplement: Supplementary file 1 [file RA-OLF-D6RA08036G-s001.pdf]

Supplementary Information (SI) for RSC Advances.
This journal is © The Royal Society of Chemistry

SUPPLEMENTARY MATERIAL

Molecularly Engineered Aspartic Acid-Intercalated Graphene Oxide-based Membranes for Ultrafast Removal of Dyes and Heavy Metal Salts

Gada Hussain[1], Sajid Hussain Siyal[2,3], Ashique Hussain Jatoi[4], Khalid Hussain Thebo[5*], Akbar Ali[6], Ahmed Nadeem[7], Gamal A. Shazly[7]

[1]School of Material Science & Engineering, Beijing University of Chemical Technology, Beijing 100029, China

[2]Széchenyi István University, Egyetem tér 1, 9026, Győr, Hungary

[3]Department of Metallurgy and Materials, Dawood University of Engineering and Technology, Karachi, Pakistan

[4]Department of Chemistry, Shaheed Benazir Bhutto University, Shaheed Benazirabad (67480), Pakistan

[5]Department of Chemistry, Mirpur University of Science and Technology, Mirpur azad Jammu and Kashmir, Pakistan

[6]MIIT Key Laboratory of Critical Materials Technology for New Energy Conversion and Storage, State Key Laboratory of Urban Water Resource and Environment, School of Chemistry and Chemical Engineering, Harbin Institute of Technology, Harbin 150001, PR China

[7]Department of Pharmaceutics, College of Pharmacy, King Saud University, Riyadh 11451, Saudi Arabia.

Corresponding Authors Email: khalidthebo@yahoo.com*

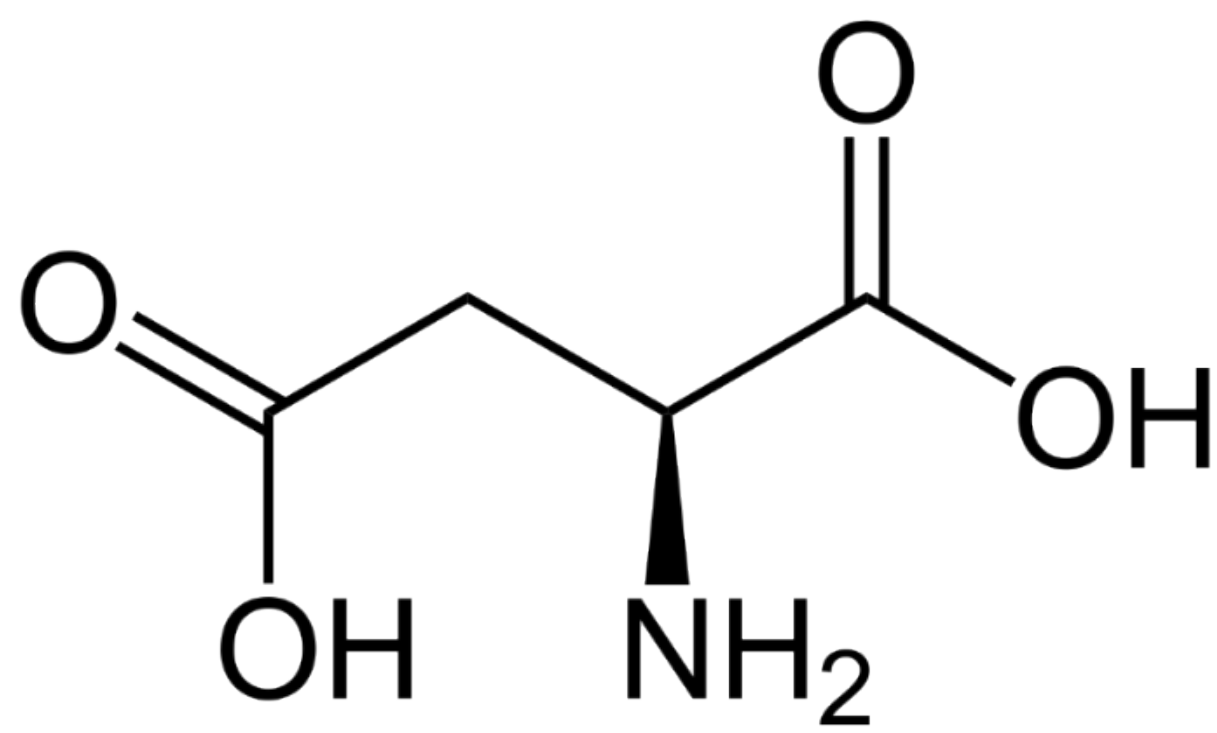

Fig. S1. Molecular structure and functionalities of Aspartic acid

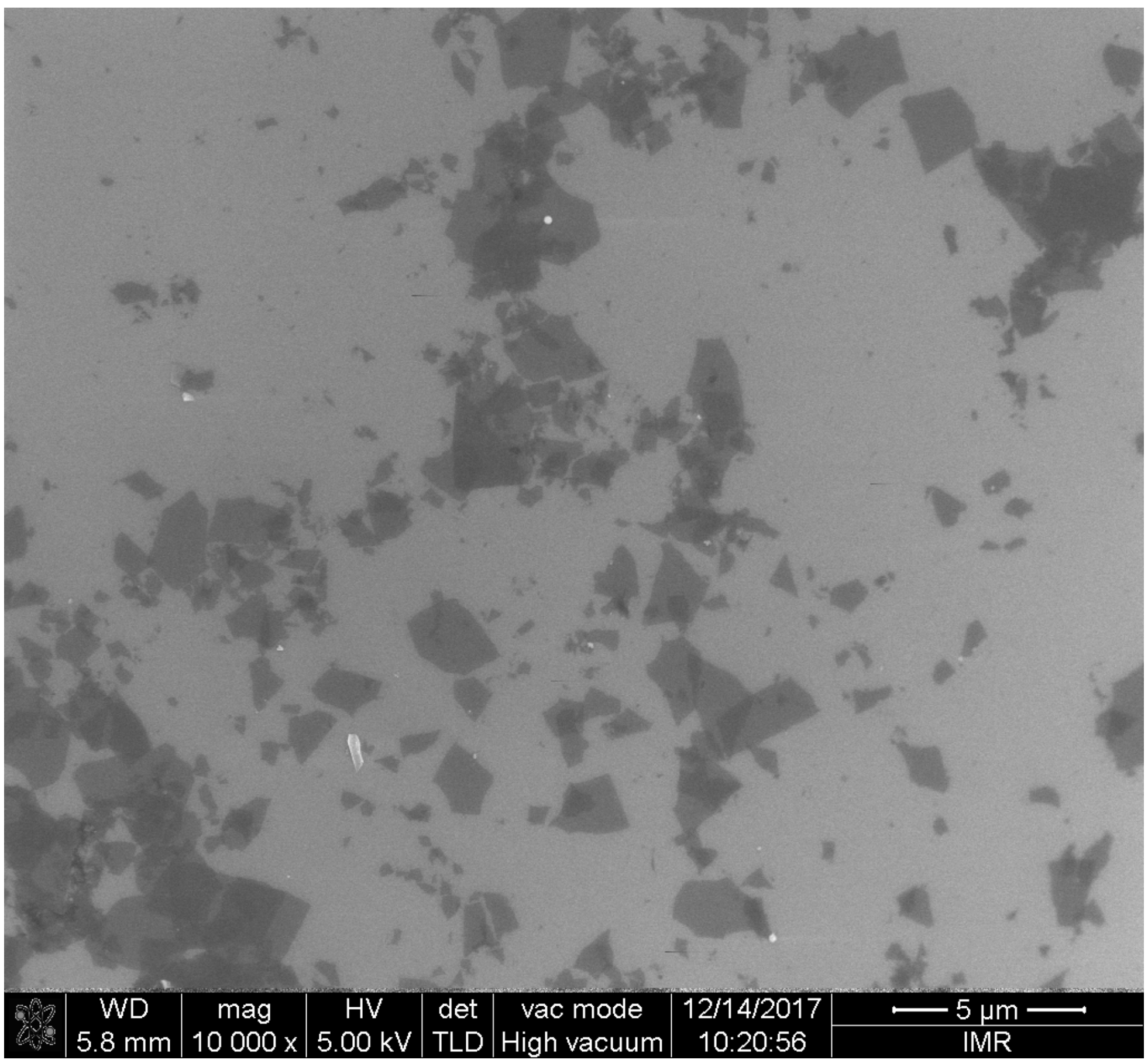

Fig. S2. Surface SEM image of GO nanosheets

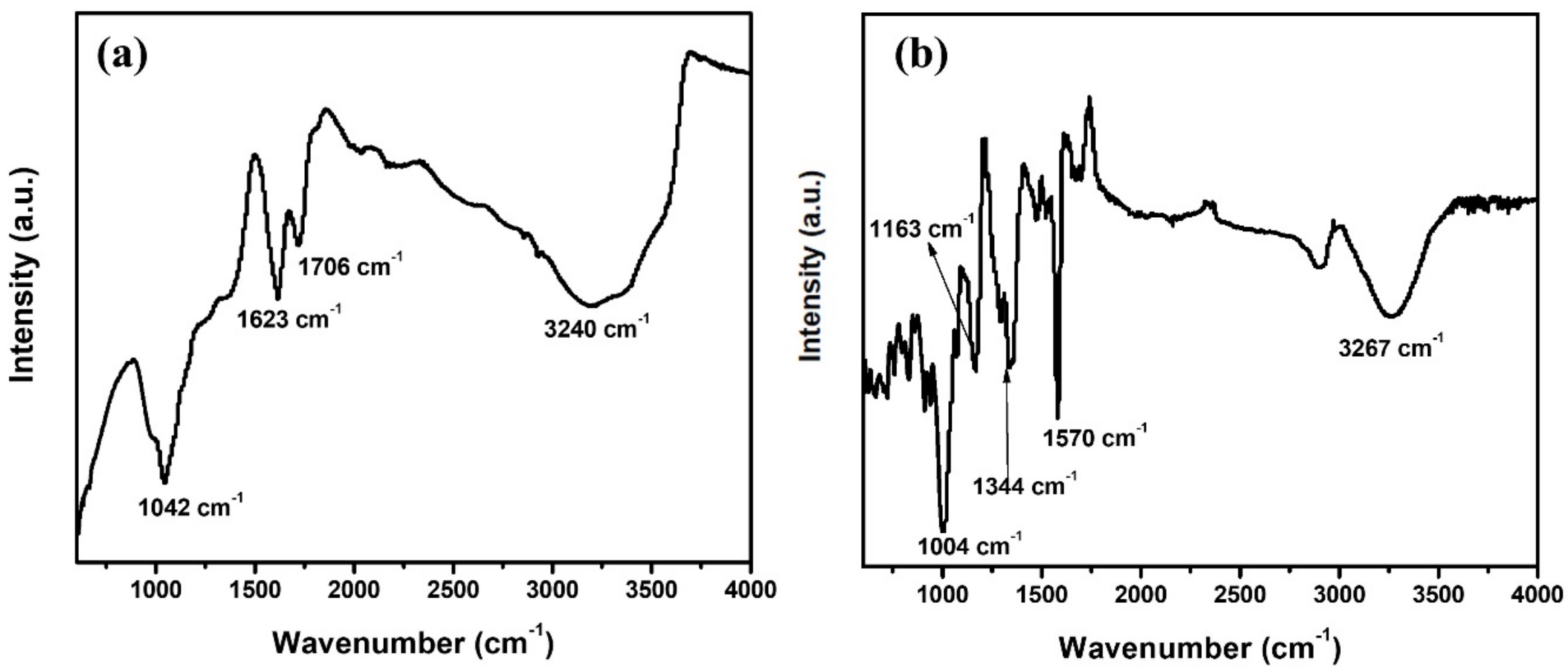

Fig. S3. FTIR spectra of (a) GO and (b) AA@GO composite membranes respectively.

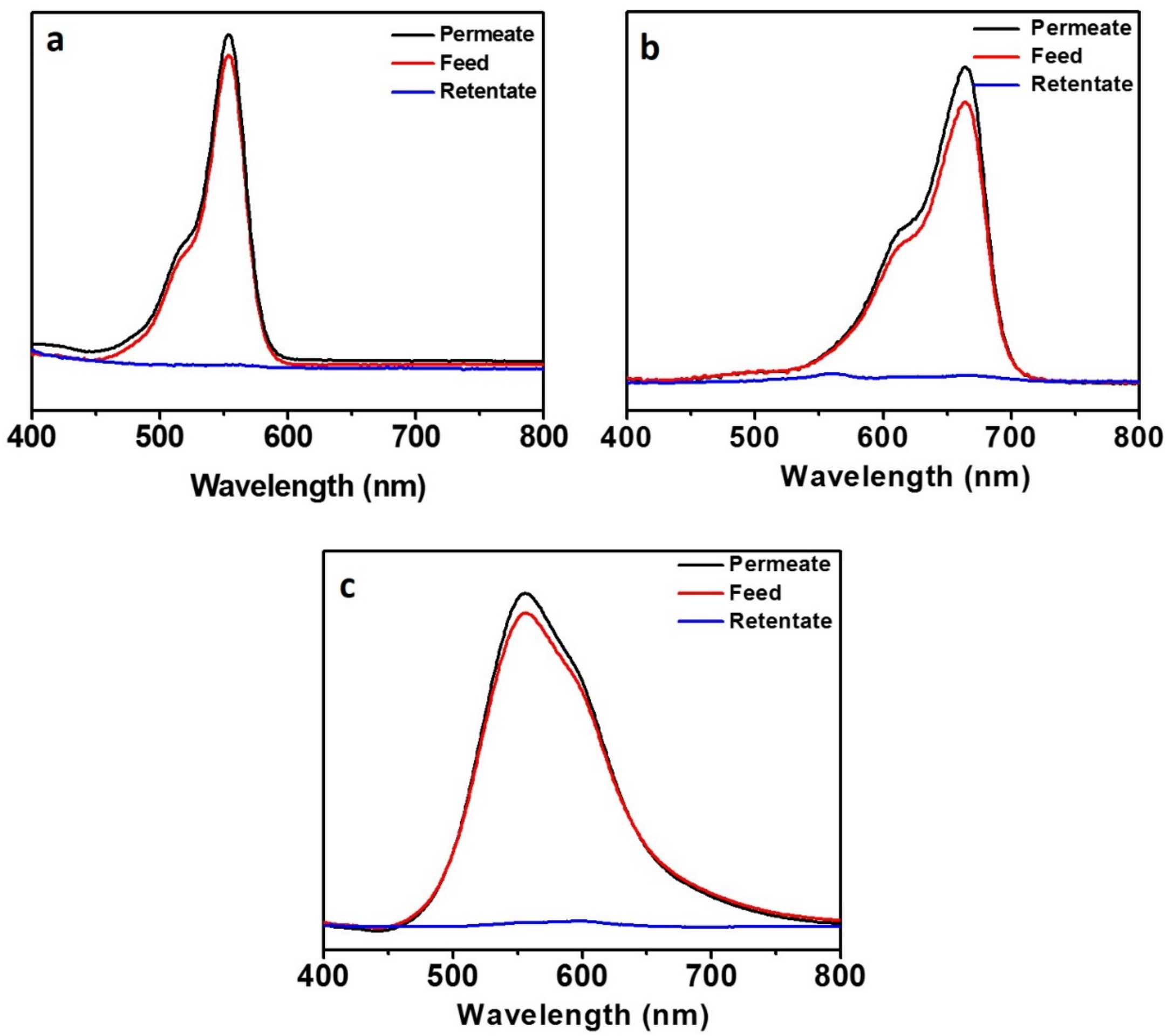

Fig. S4. UV-Vis spectra of the feed, retentate and permeate of (a) RB, (b) MLB and (**c**) EB feed solution through AA@GO composite membrane (~1330 ± 10 nm).

Table S1. The water permeance and separation efficiency of AA@GO composite membrane with different ratio of aspartic acid for separation of dyes.

GO Amount (g)	AA amount (g)	RB (100 ppm)		MLB (100 ppm)		EB (100 ppm)	
		Rej (%)	Permeance ($L\ m^{-2}\ h^{-1}\ bar^{-1}$)	Rej (%)	Permeance ($L\ m^{-2}\ h^{-1}\ bar^{-1}$)	Rej (%)	Permeance ($L\ m^{-2}\ h^{-1}\ bar^{-1}$)
0.15	0.15	99.9	730±10	99.8	800±10	98.5	815±10
0.15	0.30	100	528±10	100	513±10	100	520±10
0.15	0.50	100	312±10	100	320±10	100	335±10
0.15	0.70	100	90±10	100	105±10	100	110±10

Table S2 Filtration efficiency of pristine GO membranes against dyes and heavy metal ions.

Feed solution	Molar Mass (g/mol)	Concentration	Pristine GO membranes (320±10 nm)	
			Rejection (%)	Permeance (L m^{-2} h^{-1} bar^{-1})
RB	479.02	100 ppm	96.1	13±1
EB	960.80	100 ppm	98.2	9±1
MLB	319.85	100 ppm	95.1	14±1

Table S3. Heavy metal salt separation through AA@GO-0.15% composite membranes with different thickness.

Thickness	$AsCl_3$ (50 ppm)		$CdCl_2$ (50 ppm)		$Pb(NO_3)_2$ (50 ppm)	
	**Rej**	**Perm**	**Rej**	**Perm**	**Rej**	**Perm**
300±10	75±1	1520±10	66±1	1635±10	77±1	1430±10
680±10	82±1	920±10	81±1	975±10	84±1	667±10
970±10	92±1	452±10	89±1	525±10	92±1	175±10
1330 ±10	99.7	76±5	98.9	115±5	99.9	77±5

Abbreviation: Rej; rejection, Perm; permeance. Rejection measured in % and permeance is in L m^{-2} h^{-1} bar^{-1}.

Table S4: Benchmarking of graphene -based membranes for separation of dyes separation.

**Types of Membranes**	**Fabrication Method**	**Membrane structure[a]**	**Types of feed / Conc.**	**Permeance** (LMHB)	**Rejection (%)**	**Ref.**
GO hybrid lamellar membranes	Vacuum filtration	-	MLB	26.0	99.9	[1]
NSC-GO/PC	Vacuum filtration	l = 2.03 μm	EB (15 mM)	695	83.5	[2]
			RB (1 mM)	279	87	

GO/MB composite	Vacuum filtration	-	RB	7.67	82.56	[3]
cGO/SAA	Vacuum filtration	280 ± 10	MB	980 ± 10	98	[4]
Base-refluxing reduced GO/PVDF	Vacuum filtration	l = 22	MB (0.02 mM)	21.8	99.2	[5]
TMC cross-linked GO/PSF	Layer-by-layer	l = 5–50 layers	MB (7.5 mg L)	8–27.6	46–66	[6]
GO–SWCNT/AAO	Vacuum filtration	d = 1.8 nm	RB (15 mg L^{-1})	710	97.4	[7]
Porous GO/PC	Vacuum filtration	l = 1 μm	EB (10 mM)	191<br>-	99	[8]
GO/FLG/ deoxycholate membrane	Spray-coating	T=26–33 nm	RB	-<br>-	8	[9]

Abbreviation:[a] *d*: pore size; *l*: membrane thickness; GO: graphene oxide; NSC: Nanostrand channeled; TMC: 1,3,5-benzenetricarbonyl trichloride; SWCNT: Single walled CNT; AAO: Anodic Aluminum Oxide; PVDF: polyvinylidene difluoride; PAN: polyacrylonitrile; PAH: poly(allylaminehydrochloride); RB: Rhodamine B; MB: Methyl Blue; EB: Evans Blue; FLG: few layered graphene.

References

1. Zheng, K., et al., *Highly stable graphene oxide composite nanofiltration membrane.* Nanoscale, 2021. **13**(22): p. 10061–10066.
2. Huang, H., et al., *Ultrafast viscous water flow through nanostrand-channelled graphene oxide membranes.* Nature Communications, 2013. **4**: p. 2979.
3. Rawat, M., et al., *Textile dye degradation technologies with mechanistic insights, kinetic modeling, and comparative performance analysis.* Discover Environment, 2026. **4**(1): p. 508.

4. Chandio, I., et al., *Ultrafast ionic and molecular sieving through graphene oxide based composite membranes.* Desalination, 2021. **500**: p. 114848.
5. Han, Y., Z. Xu, and C. Gao, *Ultrathin Graphene Nanofiltration Membrane for Water Purification.* Advanced Functional Materials, 2013. **23**(29): p. 3693–3700.
6. Hu, M. and B. Mi, *Enabling Graphene Oxide Nanosheets as Water Separation Membranes.* Environmental Science & Technology, 2013. **47**(8): p. 3715–3723.
7. Gao, S.J., et al., *SWCNT-intercalated GO ultrathin films for ultrafast separation of molecules.* Journal of Materials Chemistry A, 2015. **3**(12): p. 6649–6654.
8. Ying, Y., et al., *In-plane mesoporous graphene oxide nanosheet assembled membranes for molecular separation.* RSC Advances, 2014. **4**(41): p. 21425–21428.
9. Morelos-Gomez, A., et al., *Effective NaCl and dye rejection of hybrid graphene oxide/graphene layered membranes.* Nature Nanotechnology, 2017. **12**: p. 1083.
